# Cold versus hot haemostasis in tonsillectomy: impact on post-tonsillectomy haemorrhage and pain perception - results from a randomized clinical trial

**DOI:** 10.1007/s00405-026-10027-z

**Published:** 2026-03-20

**Authors:** Michael F. Howitz, Mathias Barfred, Frej Juul Vilhelmsen, Waldemar Trolle, Malene Kirchmann

**Affiliations:** 1 Department of Otorhinolaryngology, Head & Neck Surgery and Audiology, Copenhagen University Hospital - Rigshospitalet, Dyrehavevej 29, 3400, Hillerød, Denmark; 2 Department of Otorhinolaryngology, Head & Neck Surgery and Audiology, Copenhagen University Hospital – Rigshospitalet, Copenhagen, Denmark; 3 Department of Clinical Medicine, University of Copenhagen, Copenhagen, Denmark

**Keywords:** Tonsillectomy, Adenoidectomy, Hot, Cold, Postoperative bleeding, Hemorrhage, Pain

## Abstract

**Purpose:**

The purpose of this study was to compare cold and hot haemostasis techniques during tonsillectomy in terms of the posttonsillectomy haemorrhage (PTH) risk and patient-reported postoperative pain. The primary objective was to compare the incidence of PTH from the cold- versus hot-haemostasis-treated tonsillar bed within 30 days. The secondary objective was to compare pain perception between the two sides.

**Methods:**

This was a randomized, interventional, single-centre, self-controlled clinical trial that included adolescents (aged ≥ 12 years) who underwent tonsillectomy at Nordsjællands Hospital, Denmark. Patients with malignancies, peritonsillar abscess, or coagulopathies as well as those treated with anticoagulants were excluded. Each patient received cold haemostasis (cold steel dissection and knot ligation with compression) on one tonsillar bed and hot haemostasis (cold steel dissection and bipolar diathermy, 15 W) on the contralateral side; with this method, patients served as their own controls.

**Results:**

Among the 381 included patients, 188 (49%) completed each protocol (cold/hot). Primary PTH (≤ 24 h after surgery) occurred in 12 patients (6.4%), with 6 cases of bleeding only from the cold-treated side, 3 from both sides and in 3 cases laterality was unknown (p ≤ 0.0014; OR 13.0; 95% CI 1.5–113). Secondary PTH occurred in 54 patients (29%), and of these, 33 were managed at home and 21 were admitted. Among the admitted patients, 12 bled only from the hot-treated side and 4 from the cold-treated side (p = 0.049; OR 0.33; 95% CI 0.093–0.994). More patients reported greater pain on the hot-treated side on days 12 (81%; 95% CI 73–88%) and 30 (79%; 95% CI: 65–90%) than on the cold-treated side.

**Conclusion:**

Compared with hot haemostasis, cold haemostasis is associated with a greater risk of primary PTH but significantly lower risks of secondary PTH and postoperative pain. Cold haemostasis was feasible in 49% of the patients.

**Trial registration:**

Regional Ethics Committee: H-20036864. ClinicalTrials.gov Identifier: NCT05161754.

## Introduction

Tonsillectomy remains a cornerstone procedure in otorhinolaryngology, with indications including recurrent tonsillitis, chronic tonsillitis, sleep-disordered breathing, and peritonsillar abscess. Despite its widespread use, complications such as post-tonsillectomy haemorrhage (PTH) pose significant clinical concerns [1–6].

In Denmark, 5000–7000 tonsillectomies are performed each year, most often as same-day surgery, and thus patients have two fragile tonsillar beds that need to heal in the following weeks while at home [3]. Pain is inevitable, and bleeding episodes are not uncommon and are particularly problematic because of the abrupt onset after patients have been discharged from the hospital. PTH is often classified as primary PTH (≤ 24 h after surgery) or secondary PTH (> 24 h after surgery).

Several surgical techniques have been tried to reduce the operation time and perioperative and postoperative bleeding. However, despite all the good intentions of newer and faster procedures, register- and population-based intervention studies have reported a lower risk of secondary PTH following the use of traditional cold steel instruments alone [7, 8]. Electrocautery for haemostasis was first used in surgery in 1926; however, it was not until the 1980 s that the introduction of devices such as the suction coagulator and the “electrocautery” blade alongside nonflammable anaesthesia agents became common in tonsillectomy [9, 10]. A literature review from 2018 revealed that electrocautery during tonsillectomy is effective at reducing perioperative bleeding and decreasing surgical procedure time; however, electrocautery may increase the risk of secondary PTH due to thermal trauma to surrounding tissues in the tonsillar bed, which likely impairs the healing phase [11].

The technical change from cold to hot tonsillectomy occurred gradually and was tested only in smaller randomized clinical trials with 21 to 104 patients in each cohort during the 1980 s and 1990 s [12].

This trial evaluates the impact of cold versus hot haemostasis in a randomized self-controlled setting to address this hypothesis and aims to optimize patient outcomes through evidence-based recommendations.

## Methods

### Study design

This interventional, randomized, two-armed self-controlled trial included adolescents (aged ≥ 12 years) undergoing tonsillectomy at Nordsjællands Hospital, Denmark, was approved by the regional ethics committee (H-20036864) and was conducted in accordance with the Declaration of Helsinki. The study was designed to adhere to the SPIRIT and CONSORT guidelines. The study protocol was published previously and was followed throughout the case inclusion period starting in March 2022 and ending in November 2024 [13].

### Inclusion criteria

Patients referred for bilateral tonsillectomy were eligible for inclusion. The criteria for tonsillectomy due to recurrent acute tonsillitis were at least five episodes of tonsillitis within one year or three episodes of tonsillitis per year in two consecutive years [14] Chronic tonsillitis is characterized by persistent tonsillitis, irritation due to tonsil stones and/or bad breath/taste. The exclusion criteria were the presence of peritonsillar abscess or parapharyngeal abscess, suspected tonsil malignancy, treatment with anticoagulant medication and known coagulopathy. Patients' who had undergone previous drainage of a peritonsillar abscess were included.

### Randomization and intervention details

Eligible patients who agreed to participate were randomized using REDCap software with permuted blocks to undergo *cold dissection and cold haemostasis* on one side and *cold dissection and hot haemostasis* on the other side.

*Cold dissection and cold haemostasis* were defined as dissection using the cold steel technique followed by the use of a resorbable Vicryl knot (Endoloop) around the lower tonsil pillar and compression of the tonsillar bed with a peanut gauze ball for five minutes (if bleeding occurred after five minutes of compression, the surgeons were instructed to repeat compression up to two times for five minutes each time); furthermore, packs, ties, sutures, and 1–2 mg/ml adrenaline compression/infiltration were used to stop any perioperative bleeding in the tonsillar bed. If bleeding continued after compression was performed 3 times for 5 min each time or if bleeding exceeded 200 ml during the procedure, the surgeon was allowed to continue with hot haemostasis, and the haemostasis was hereafter categorized as “hot”.

*Cold dissection and hot haemostasis* were defined as dissection using the cold steel technique followed by hot haemostasis with bipolar cautery (15 watts). No monopolar or other haemostatic surgical devices were used in this study.

In Denmark, tonsillectomy is among the first procedures learned in the field of ENT, and therefore, many tonsillectomies are performed by less experienced surgeons. We have not deviated from this framework. However, all the participating surgeons had performed at least ten tonsillectomies or were supervised by an experienced ear, nose, and throat surgeon.

If the surgeon could not achieve haemostasis without diathermy, the surgeon provided this information during the operation and described what was performed in the patient’s file and in the case report in REDCap after the procedure. Those patients were not excluded from the study or from further data collection but were excluded from the per-protocol analysis of the interventional randomized self-controlled trial comparing cold and hot haemostasis. The option to consult a senior colleague (ear, nose, or throat specialist) was always available. Blood was suctioned from the operative field, and the bleeding volume was measured in ml perioperatively and postoperatively using standard equipment.

By this design, every patient was intended to be treated with both interventions and thus serve as his or her own control. The surgeons were obviously not blinded to the surgical procedure, and since the surgical procedure was documented in the patient files, the patient was also not blinded to the surgical procedure.

### Postoperative care

Patients were observed for four hours after surgery in the ward and were then discharged in most cases; this was the same observation time and procedure used before the start of the study. Postoperative information regarding the risk of oral bleeding, along with instructions to call emergency services if bleeding did not stop after approximately 15 min of the application of ice in the oral region, were routinely provided. Pain relievers, including paracetamol, ibuprofen, and for some patients, additional morphine, were also prescribed. In Denmark, antibiotics are not prescribed either before or after tonsillectomy, and no dietary restrictions are recommended following the surgery.

When at home, the patients received an automatically generated REDCap e-mail on days 6, 12 and 30 after tonsillectomy, which gave them unique access to a REDCap questionnaire that contains several questions: Has bleeding been observed? Was the bleeding from the left or the right side? What analgesic medications have been consumed? The questionnaire also includes a question about pain perception using a visual analogue scale ranging from 1–10 [13].

### Outcome measures

#### Primary outcome

PTH incidence in the cold and hot tonsillar beds within 30 days after surgery was reported via REDCap surveys on days 6, 12, and 30 and in the electronic journal. PTH is reported as either primary (≤ 24 h) or secondary (> 24 h). Patients with primary PTH may also have been included in the count of patients with secondary PTH.

#### Secondary outcome

Pain severity was measured on a 10-point linear numeric scale.

### Sample size calculation

On the basis of an estimated 12% PTH risk for adolescents (aged ≥ 12 years), a sample size of 175 patients was calculated to achieve 80% power at a 5% significance threshold [13].

### Data collection and analysis

Data were collected and managed using REDCap electronic data capture tools hosted at Region Hovedstaden [15, 16]. Data analysis was performed using R software (v4.1.3). The median is reported if not otherwise stated, and the interquartile range (IQR) is in brackets. Continuous variables were compared between the two independent groups using the Mann–Whitney U (Wilcoxon rank-sum) test. Categorical variables were compared using Pearson’s chi-square test when expected cell counts were ≥ 5 and Fisher’s exact test when expected counts were < 5. Paired proportions (cold versus hot tonsillar bed within the same patient) were analysed using the Mid-P McNemar test. A two-sided *p* value < 0.05 indicated statistical significance. The data were analysed per-protocol and not as intention-to-treat, as the stated primary intention of the study was to analyse the two surgical techniques under ideal conditions in the same individual. Performing an intention-to-treat analysis in this study would bias the results towards the null hypothesis. If a tonsillar bed initially assigned to cold haemostasis was subsequently converted to hot haemostasis, an intention-to-treat approach would still analyse it as part of the intended cold haemostasis group. To be transparent, we present in Tables 2 and 3 data on intention-to-treat patients (overall), per-protocol patients (cold/hot) and patients treated with electrocautery in both tonsillar beds (hot/hot).

## Results

### Participant characteristics

**Fig. 1:**
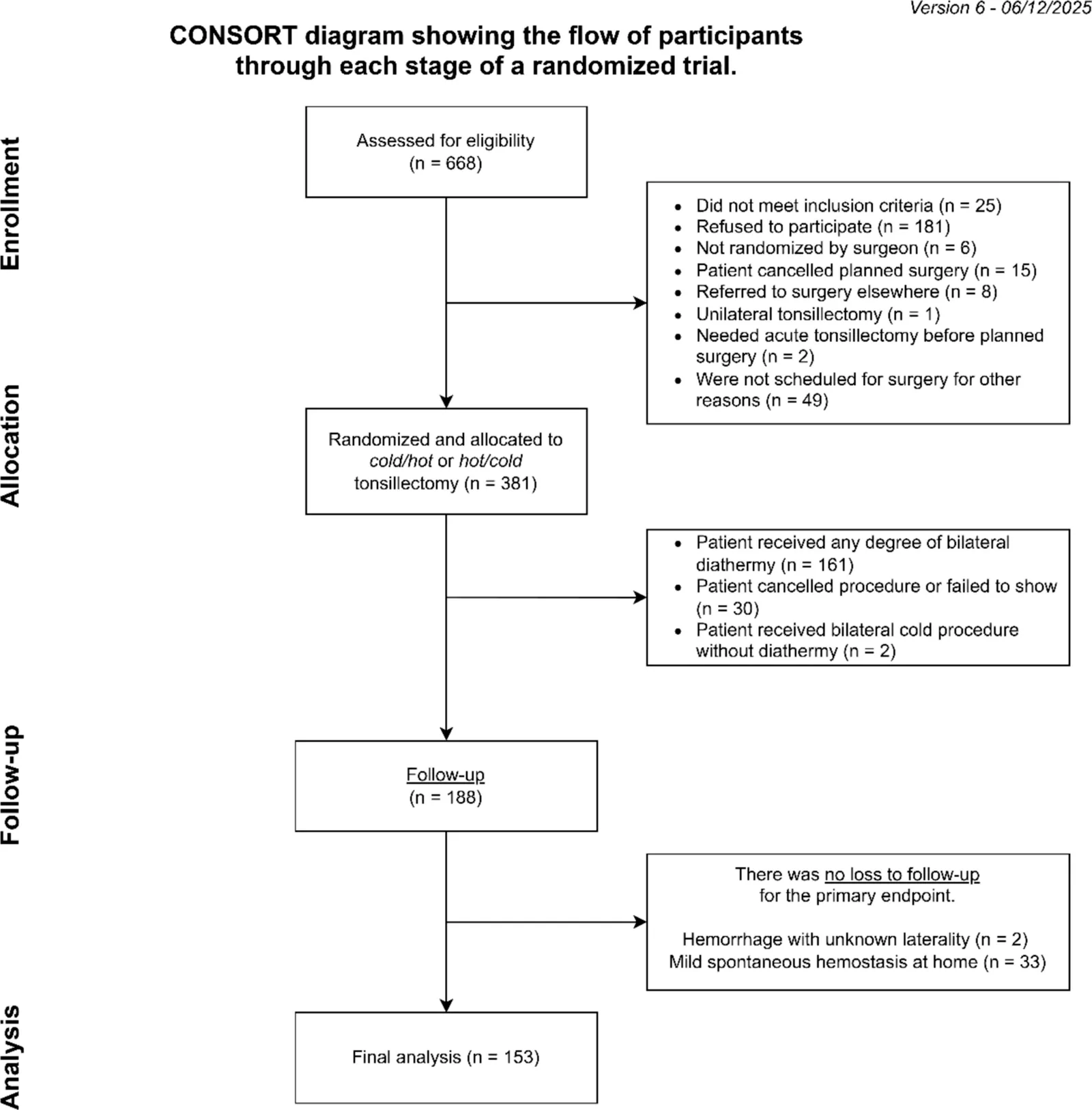
CONSORT diagram on the flow of patients following the study setup. Among the 32 patients for whom surgeons were unable to adhere to the research protocol, the reasons were as follows: 26 patients had cancelled their surgeries before operation day, four patients failed to appear on the day of the operation and were dismissed, and two patients underwent the procedure with cold dissection and haemostasis on both sides

During the 2½-year inclusion period, 668 adolescents (aged ≥ 12 years) were scheduled to undergo elective tonsillectomy, and of these, 64% were females, and the median age was 22 years (interquartile range (IQR) 18–32). Among the patients, 287 were excluded for various reasons (Fig. 1). In all, 381 patients provided written consent and were enrolled and randomised according to the protocol. However, in 32 (8.4%) patients, the surgeon could not adhere to the protocol, and in 161 (42.3%) patients, the procedure was converted to hot haemostasis in the intended cold tonsillar bed, which resulted in 188 (49.3%) patients treated according to the protocol (Fig. 1).

The indications for tonsillectomy were recurrent acute tonsillitis (41%), tonsil stones (24%), chronic tonsillitis (22%), bad breath (17%), previous peritonsillar abscess (9%), sleep apnoea (3%) and other (3%). The cohort consisted of adolescents and young adults without comorbidities, and medication used was limited to contraceptives in most patients.

### Surgeon characteristics

In all, 56 different surgeons performed 381 tonsillectomies. Each surgeon performed between one and 25 tonsillectomies, with a median number of four. Self-rated surgical experience in tonsillectomy ranged from 34% (who had previously performed 0–25 tonsillectomies) to 25% (who had performed 26–50 tonsillectomies), to 34% (who had performed > 50 tonsillectomies), while experience with tonsillectomy was unknown for 7% of surgeons. All tonsillectomies were performed during the day (8 am to 5 pm).

### Primary PTH

Among the 188 patients who underwent cold and hot tonsillectomy, 12 had primary PTH (≤ 24 h) (6.4%). Among these patients, bilateral haemorrhage occurred in three, haemorrhage occurred on the cold-treated side in 6 patients, and no instances of bleeding occurred on the hot-treated side; laterality was unknown in three patients. The paired odds ratio was 13.0 (95% CI 1.5–113; *p* = 0.014). Among the 12 patients with primary PTH, bleeding stopped without intervention in nine patients, bleeding stopped by manual compression with peanut gauze on the tonsillar bed in two patients, and one patient required a heamostasis surgery. Postoperative hospital stay was prolonged to the next day in three patients. Among the 12 patients with primary PTH, six reported secondary PTH, five had spontaneous haemostasis while at home, and one patient returned to the hospital and required another surgery.

### Secondary PTH

Among the 188 patients who underwent cold and hot tonsillectomy, 54 (29%) experienced secondary PTH. Among these 54 patients, PTH stopped spontaneously at home (61%) in 33 patients. For 16 of the 33 patients whose PTH resolved at home, the laterality of bleeding was unknown. When all cases of secondary PTH were analysed, we did not find a significant difference between the cold- and hot-treated groups (*p* = 0.612).

Among the 21 patients (11%) who were admitted to the hospital for secondary PTH, three experienced bleeding from both tonsillar beds, 12 from the hot-treated tonsillar bed, and four from the cold-treated tonsillar bed, while the laterality was unknown in two patients (*P* = 0.049) (Table 1). When all patients with secondary PTH and known bleeding laterality who were admitted to the hospital (19 of the 153 eligible patients) were analysed, we found a significant difference between the cold- and hot-treated tonsillar beds, with an odds ratio of 0.33 (95% CI 0.093–0.994) (Table 1). The median time to secondary PTH was six days (IQR 4,8) after tonsillectomy.

**Table 1:** Secondary haemorrhage among patients who had one tonsil removed with cold haemostasis and one tonsil removed with warm haemostasis

	Hemorrhage after hot procedure		Total
	No	Yes	
Hemorrhage after cold procedure			
No	134 (88%)	12 (7.8%)	146 (95%)
Yes	4 (2.6%)	3 (2.0%)	7 (4.6%)
Total	138 (90%)	15 (9.8%)	153 (100%)

McNemar’s Chi-squared test, *p* = 0.046

Paired samples. Mid-P McNemars test: *p* = 0.0490, OR 0.333 (95%CI 0.0930, 0.9944) *(Mid-P Exact Clopper-Pearson CI)*

Not including 2 cases with unknown laterality of hemorrhage

### Pain

**Fig. 2:**
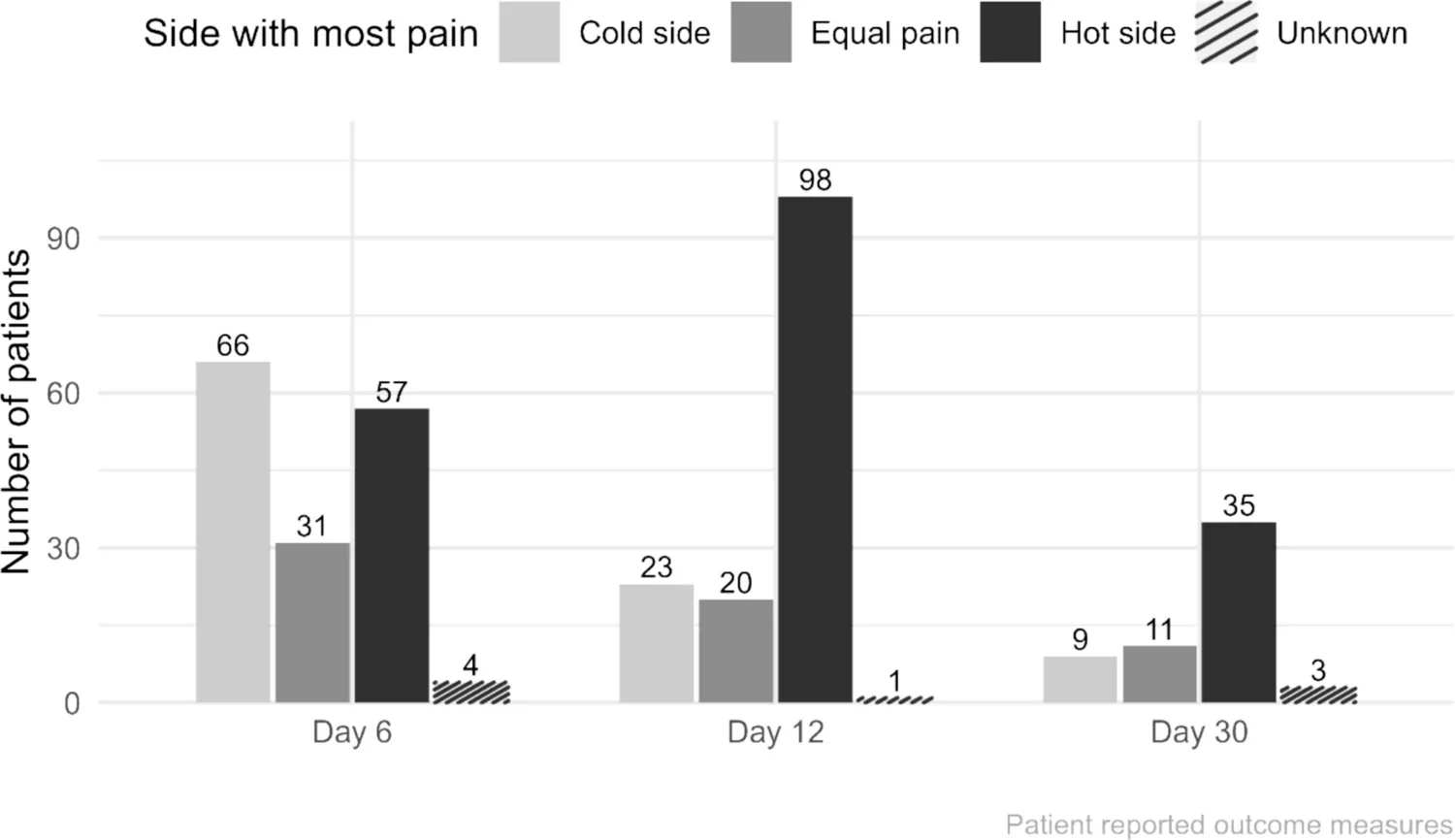
Distribution of the side with the greatest pain on postoperative days 6, 12, and 30 following tonsillectomy, as reported by the respondents

We found no significant difference in most patient-reported laterality of pain on day six postsurgery, as 46% reported greater pain on the hot-treated side (*p* = 0.063; 95% CI 37–56%). However, on day 12, significantly more patients reported an increased sensation of pain on the hot-treated side than on the cold-treated side (81%; 95% CI 73–88%). The same was observed for day 30 (79%; 95% CI 65–90%) (Fig. 2) (Table 2).

**Table 2:** Surgical characteristics, primary and secondary posttonsillectomy haemorrhage and pain perception

Characteristics	Statistic	Overall *N* = 358	Type of tonsillectomy		*p*-value^*1*^
			Cold/Hot *N* = 188	Hot/Hot *N* = 169	
Surgery					
Concurrent adenotomy	n (%)	44 (12.4%)	18 (9.6%)	26 (15.6%)	0.11
Perioperative bleeding (mL)	Median (Q1, Q3)	20 (10, 50)	20 (10, 30)	25 (15, 60)	< 0.001
Surgeon perceived difficulty of case					< 0.001
* Easy*	n (%)	169 (49.0%)	104 (57.1%)	65 (39.9%)	
* Average*	n (%)	119 (34.5%)	64 (35.2%)	55 (33.7%)	
* Difficult*	n (%)	35 (10.1%)	7 (3.8%)	28 (17.2%)	
* Unknown*	n (%)	22 (6.4%)	7 (3.8%)	15 (9.2%)	
Surgery time including hemostasis (minutes)	Median (Q1, Q3)	50 (38, 64)	45 (35, 57)	59 (44, 72)	< 0.001
Additional time as a result of research procedure (minutes)	Median (Q1, Q3)	5 (0, 12)	5 (0, 10)	10 (5, 15)	< 0.001
Hemorrhage					
Primary hemorrhage	n (%)	29 (8.1%)	12 (6.3%)	17 (10.1%)	0.25
Secondary hemorrhage	n (%)	110 (30.7%)	54 (28.6%)	56 (33.1%)	0.36
Episodes of hemorrhage	Median (Q1, Q3)	2 (1, 3)	1 (1, 2)	2 (1, 3)	0.024
Days until first secondary hemorrhage	Median (Q1, Q3)	6.0 (4.0, 7.0)	6.0 (4.0, 8.0)	6.0 (4.0, 7.0)	0.70
Type of secondary hemorrhage					0.26
* Return to operating theater*	n (%)	11 (3.1%)	5 (2.6%)	6 (3.6%)	
* Admitted to hospital*	n (%)	41 (11.5%)	16 (8.5%)	25 (14.8%)	
* Spontaneous hemostasis at home*	n (%)	58 (16.2%)	33 (17.5%)	25 (14.8%)	
* No hemorrhage*	n (%)	248 (69.3%)	134 (71.3%)	113 (66.9%)	
Pain					
Self-reported pain on day 6 (NRS)	Median (Q1, Q3)	6 (5, 8)	6 (5, 8)	6 (5, 8)	0.95
Self-reported pain on day 12 (NRS)	Median (Q1, Q3)	3 (2, 5)	3 (2, 4)	3 (2, 5)	0.24
Self-reported pain on day 30 (NRS)	Median (Q1, Q3)	1 (1, 2)	1 (1, 2)	1 (1, 2)	0.85
Self-reported pain requiring opioids on day 6	n (%)	139 (38.8%)	88 (46.6%)	51 (30.2%)	0.002
Self-reported pain requiring opioids on day 12*	n (%)	29 (8.1%)	14 (7.4%)	15 (8.9%)	0.70

^1^ Fisher’s exact test; Wilcoxon rank sum test

There we no patients requiring opioids on day 30

When we compared those who underwent surgery according to the protocol (cold/hot) with those who underwent bilateral electrocautery, we found no clinically significant differences between the cold/hot and hot/hot groups. However, significantly more patients in the cold/hot group used paracetamol, ibuprofen, and opioids as analgesics according to the questionnaire given on day six (Table 3). According to the questionnaire given on day 12, for the three different analgesic types, the cold/hot and hot/hot groups used analgesics at comparable rates. By day 30, analgesic use was minimal (paracetamol < 3%, NSAIDs < 2%, and opioids 0%), with no significant differences in overall consumption (Table 3). The median pain scores did not significantly differ between the cold/hot group and the hot/hot group, as they decreased from six on day six to three on day 12 and one on day 30 (Table 3).

**Table 3:** Use of postoperative analgesics on days 6, 12 and 30 after tonsillectomy

Characteristics	Statistic	Overall *N* = 358	Type of tonsillectomy		*p*-value^*1*^
			Cold/Hot *N* = 188	Hot/Hot *N* = 169	
Use of paracetamol on day 6	n (%)	262 (73%)	151 (80%)	111 (66%)	0.003
Use of paracetamol on day 12	n (%)	178 (50%)	94 (50%)	84 (50%)	> 0.99
Use of paracetamol on day 30	n (%)	7 (2.0%)	4 (2.1%)	3 (1.8%)	> 0.99
Use of NSAID on day 6	n (%)	227 (63%)	130 (69%)	97 (57%)	0.028
Use of NSAID on day 12	n (%)	128 (36%)	71 (38%)	57 (34%)	0.51
Use of NSAID on day 30	n (%)	5 (1.4%)	3 (1.6%)	2 (1.2%)	> 0.99
Use of opioids on day 6	n (%)	139 (39%)	88 (47%)	51 (30%)	0.002
Use of opioids on day 12	n (%)	29 (8.1%)	14 (7.4%)	15 (8.9%)	0.70
Use of opioids on day 30	n (%)	0 (0%)	0 (0%)	0 (0%)	> 0.99
Use of other painkillers day 6	n (%)	1 (0.3%)	0 (0%)	1 (0.6%)	0.47
Use of other painkillers day 12	n (%)	1 (0.3%)	0 (0%)	1 (0.6%)	0.47
Use of other painkillers day 30	n (%)	0 (0%)	0 (0%)	0 (0%)	> 0.99
Use of 'unknown' painkillers day 6	n (%)	1 (0.3%)	1 (0.5%)	0 (0%)	> 0.99
Use of 'unknown' painkillers day 12	n (%)	0 (0%)	0 (0%)	0 (0%)	> 0.99
Use of 'unknown' painkillers day 30	n (%)	0 (0%)	0 (0%)	0 (0%)	> 0.99

^1^ Fisher’s exact test

### Surgical characteristics

Using a three-level rating scale, the surgeons independently determined that most patients were “easy”; however, compared with the cold/hot group, the hot/hot tonsillectomy group was rated significantly more difficult (Table 2).

The median perioperative bleeding volume was significantly greater in the hot/hot group than in the cold/hot group (10 ml, 95% CI 5–15; *p* < 0.001) (Table 2). The greatest volume of perioperative bleeding was observed in two patients whose bleeding volume was 300 ml. One patient remained in the cold/hot group and had no PTH, and the other patient was converted to the hot/hot group because the knot around the tonsil pillar fell off during surgery. The latter patient experienced PTH. In four patients, the surgeon used an additional suture in the tonsillar bed to stop the perioperative bleeding.

The median surgical duration, including haemostasis, in the cold/hot group was 45 min (range, 35–57), and the median additional reported surgical time due to inclusion of the cold procedure in one tonsillar bed was estimated by the surgeons to be five minutes (range, 0–10) (Table 2). The median surgery time was significantly longer in the hot/hot group (59 min, IQR 44–72) than in the cold/hot group (45 min, IQR 35–57). The estimated median difference in surgical time was 12 min (95% CI 8–16, p < 0.001). The dataset was too limited to allow stratification according to surgeon experience.

### Overall PTH

The overall prevalence of primary PTH was 8.1% (29/358), and that of secondary PTH was 30.7% (110/358). No significant difference in primary or secondary PTH was detected between the cold/hot and hot/hot groups. However, compared with patients in the cold/hot group, each patient in the hot/hot group had significantly more bleeding episodes (median 2 vs. 1; *p* = 0.024). The estimated median difference was approximately one additional episode (95% CI 0–1). Furthermore, a nonsignificant tendency towards more serious bleeding episodes was observed in the hot/hot group, which was associated with increased admission to the hospital (15% versus 8.5%) and a return to the operating theatre (3.5% versus 2.4%) (Table 2). The median time until the occurrence of secondary PTH was six days (Q1, Q3, 4 to 8), and no difference was found between the two groups (Table 2).

### Adverse events

Apart from bleeding, no other severe adverse events were reported or observed when the electronic patient records were examined.

## Discussion

In this study, we found that significantly more patients experienced primary PTH from the cold-treated tonsillar bed and that significantly more patients experienced secondary PTH from the hot-treated tonsillar bed. Additionally, significantly more patients reported postoperative pain from the hot-treated tonsillar bed at days 12 and 30 after tonsillectomy.

The overall prevalence of primary PTH among 358 tonsillectomies was 8.1%, while the prevalence of secondary PTH was 30.7%, and of the patients, 52.7% (58/110) reported self-limiting bleeding episodes at home. When we deducted the 16% of patients with PTH with spontaneous haemostasis at home, we found an overall secondary PTH prevalence of 15%; these patients were admitted to the hospital, and 3% (11/358) returned to the operating theatre.

It was possible to perform cold haemostasis in one tonsillar bed in only 49% of the patients, and the median estimated additional surgery time for the cold treatment of one tonsillar bed was 5 min.

### Post-tonsillectomy haemorrhage

Our finding of an overall primary PTH rate of 8% aligns with published rates ranging from 0.5% to 10%, which depend on the definitions and surgical techniques used [3–5]. The lack of a significant difference in primary PTH between the cold/hot and hot/hot groups suggests that the perioperative haemostasis technique used is comparable within the first 24 h. However, we viewed the significantly greater perioperative bleeding volume observed in the hot/hot group, coupled with the surgeons' report that hot/hot tonsillectomies were more difficult, to be natural confounders of the protocol. Each surgeon was instructed to provide compression three times for five minutes each time, adrenalin injection, and/or suture to obtain haemostasis before they resorted to bipolar diathermy. Consequently, the patients in the hot/hot group in this setup inherently require a longer surgery time and are considered more technically challenging cases. When the cold/hot versus hot/hot groups were compared, to a considerable degree, this was a comparison of easy versus complicated patient cases and not a comparison of surgical techniques.

The secondary PTH rate of 31% in this study was at the high end of the rates reported in the literature, which ranged from 0.5% to 33% [4–6, 13]. A previous Danish register-based study reported that hospital admitted PTH rates increased from 3 to 13% from 1994 to 2012 [3]. A Swedish study based on data from The Swedish Quality Register for Tonsil Surgery reported PTH rates of 15–20% [7]. These discrepancies likely reflect differences in study populations, surgical settings, definitions of PTH and how the data were collected. This study is sensitive to the collection of data on bleeding episodes at home as well as in the hospital setting because three patients self-reported their bleeding episodes through REDCap within the first month; the patient records were also scrutinized within one month after tonsillectomy. In terms of self-limiting PTH episodes at home, the overall rate of secondary PTH was 15%, which is higher than that reported from previously findings in Denmark [3].

### Pain perception and analgesic use

Patient-reported pain scores revealed increased use of analgesics in the cold/hot group on day six, which suggests that the initial healing phase may involve increased discomfort on the cold-treated side. Interestingly, when asked about the most painful side, patients reported the hot side on days 12 and 30 after tonsillectomy, which supports concerns that thermal trauma may exacerbate prolonged nociceptive responses and healing [17].

These findings are consistent with the literature that suggests that while electrocautery may provide efficient intraoperative haemostasis, it can lead to increased postoperative tissue damage, inflammation, and risk of secondary haemorrhage [7, 11]. Interestingly, a systematic review from 2003 revealed that all six included studies reported significantly more pain from postoperative days four to ten with electrocautery tonsillectomy [12].

### Surgical duration

In this study, the surgeons reported that the use of a self-locking suture knot and keeping one tonsillar bed cold increased the surgical time by a median of five minutes (25th and 75th percentile; 0–10 min). Fifty-six different surgeons performed the tonsillectomies, and the protocol stated that after the suture knot was set, the surgeon should perform compression of the cold tonsillar bed up to three times for five minutes each time. Forty-two percent of patients converted to hot procedures in the intention-to-treat cold tonsillar bed after the compression period, and the median operation time in the hot/hot group was 14 min longer. A retrospective study by Senska et al., in which a horizontal mattress suture was placed to adapt the faucial pillars during tonsillectomy, revealed an increase in surgery time of 8 min and a rate of postoperative haemorrhage that was decreased by 50% [18]. Elkholy reported that suturing the faucial pillars during tonsillectomy with multiple interrupted sutures increased the surgery time by five minutes [19].

A slight learning curve for setting a self-locking knot during tonsillectomy was noted. An often-encountered surgical challenge is the ability to tighten the self-locking knot around the lower tonsil pillar while simultaneously loosening the Negus tonsil forceps. Sometimes the knot fell off if was too loose or it cut through the tonsil pillar when it was too tight. When such an incident occurred, the surgeons were likely to convert the procedure to hot haemostasis. Our experience from this study is that the cold tonsillectomy technique is mastered after a few tonsillectomies.

### Clinical implications

Multiple factors that influence PTH risk have been identified in several studies, including increased age, male sex, overweight status, comorbidities, medication, smoking status, indication for tonsillectomy and surgical technique used [5–7, 11, 20–29]. Studies on surgical techniques have identified the following risk factors that increase the incidence of PTH: less experienced surgeons, removal of deeply buried and inflamed tonsils, and the use of perioperative coblation and electrocautery [3, 6].

Several studies have reported on different attempts to reduce haemorrhage and pain from tonsillectomy, including oversewing the tonsil beds, applying various supposed healing materials to the tonsillar beds, and using novel surgical instruments and postoperative care approaches, but no procedure has been demonstrated to be markedly better than conventional methods and none has gained wide acceptance [21, 27, 30, 31]. Tonsillotomy, which is mostly performed in younger children with tonsillar hyperplasia, has been shown to decrease the bleeding rate compared with that of tonsillectomy [7, 24, 26].

Despite the increasing evidence that indicates the benefit of the cold technique of tonsillectomy, its adoption has been limited in Denmark. A comparable Scandinavian study revealed that 23% of tonsillectomies in Sweden, 10% in Norway and 6% in Denmark were performed with cold techniques [32]. The national guidelines in Sweden support the use of the cold-cold technique, and the same recommendation applies in Norway on the basis of a national improvement project on the use of the tonsillectomy technique and postoperative haemorrhage [7, 8, 29, 33, 34]. A newly published systematic review including 12 randomized controlled trials with a total sample size of 1684 subjects concluded that cold steel dissection appears to be the safest method for the prevention of secondary haemorrhage [35]. Reasons for the hesitation in Denmark may be increased intraoperative bleeding, extended surgical time and increased risk of primary PTH [32].

The results of this study support the broader adoption of cold haemostasis techniques during tonsillectomy in Denmark, particularly to reduce secondary PTH risk and to shorten postoperative pain sensation from postoperative week two. While cold techniques increase surgical time and the occurrence of primary haemorrhage, this trade-off appears justifiable given the observed reduction in secondary PTH after the patient is discharged from the hospital. The implementation of a standardized protocol that prioritizes cold haemostasis could improve safety and reduce discomfort, considering that a high conversion rate to hot haemostasis is justifiable.

We expect that the average additional surgical time to perform a tonsillectomy with self-locking suture knots around both lower tonsil pillars and to keep both tonsillar beds cold will be less than 10 min if this study’s approach is adopted with 3 × 5 min of compression at most. Furthermore, according to the Swedish data, we expect that approximately three of four patients will be converted to hot haemostasis in one or both tonsillar beds during tonsillectomy [32].

A Norwegian approach revealed that structured video-based instruction, teaching, and training in cold techniques for tonsillectomy resulted in a reduction in PTH from 18% in 2017/2018 to 7% in 2020 [8].

### Strengths and limitations

This study’s key strength lies in its robust intrapatient design, which minimizes confounding factors. Randomization and the use of validated data collection tools further increase the reliability of the findings. However, the single-centre setting may limit generalizability, and variability in surgical experience, as 59% of surgeons had previously performed < 50 tonsillectomies, could have influenced outcomes despite protocol adherence. Furthermore, due to the 42% conversion rate to hot haemostasis, extrapolation from the per-protocol analysis may overestimate the treatment effect of the cold-cold tonsillectomy technique. We did not document the dominant hand of the surgeon but did not consider this to be a cause of bias because of randomization.

### Future directions

Future research should explore tonsillar bed healing, scarring, and functional outcomes following different haemostasis techniques. One hypothesis to test is whether perioperative adrenaline use in a tonsillar bed increases postintubation primary haemorrhage.

Considering this study and comparable findings in the literature, we believe that it is time to implement national guidelines to minimize the widely used practice of electrocauterization in tonsillectomy. The importance of the cold steel technique should be prioritized when residents are trained in the tonsillectomy procedure. Additionally, large multicentre trials should be advocated to confirm these findings and to assess their applicability across diverse populations and healthcare systems. The investigation of alternative techniques, such as advanced suture materials, the amount of energy delivered during electrocautery when haemostasis is performed on a vessel in an open wound, and alternative topical haemostatic agents, could further enhance surgical outcomes [24, 25].

## Conclusion

This trial demonstrated that compared with hot haemostasis, cold haemostasis, which is more technically demanding, is associated with a longer operative time and is linked to a higher risk of primary haemorrhage; moreover, cold haemostasis is associated with a significantly lower risk of secondary posttonsillectomy haemorrhage and results in less postoperative pain. Given that secondary haemorrhage poses a greater clinical and patient safety burden, our findings indicate that the overall risk–benefit profile favours cold haemostasis. We therefore support a shift towards cold haemostasis techniques in tonsillectomy to enhance both patient safety and postoperative comfort.

## Data Availability

The data supporting the findings of this study were collected and managed using REDCap electronic data capture tools at Region Hovedstaden, Denmark. The data are not publicly available due to patient confidentiality and data protection regulations. Deidentified data may be made available from the corresponding author upon reasonable request, subject to approval by Region Hovedstaden and compliance with applicable Danish data protection legislation.

## References

[1] Sarny S, Habermann W, Ossimitz G et al (2011) Tonsilar haemorrhage and re-admission: a questionnaire based study. Eur Arch Otorhinolaryngol 268:1803–1807. 10.1007/s00405-011-1541-y 21373896

[2] DA Randall HM (1998) Complications of tonsillectomy and adenoidectomy - a comparison of techniques 1998. Otolaryngol Head Neck Surg 4:375–380 10.1016/S0194-5998(98)70376-6 9450830

[3] Juul MLB, Rasmussen ER, Howitz MF (2020) Incidence of post-tonsillectomy haemorrhaging in Denmark. Dan Med J 67:A11190640 32741442

[4] Kvaerner KJ (2009) Benchmarking surgery: secondary post-tonsillectomy hemorrhage 1999–2005. Acta Otolaryngol 129:195–198. 10.1080/00016480802078101 18607926

[5] Blomgren K, Qvarnberg YH, Valtonen HJ (2001) A prospective study on pros and cons of electrodissection tonsillectomy. Laryngoscope 111:478–482. 10.1097/00005537-200103000-00018 11224779

[6] Gysin C, Dulguerov P (2013) Hemorrhage after tonsillectomy: does the surgical technique really matter? ORL J Otorhinolaryngol Relat Spec 75:123–132. 10.1159/000342314 23978795

[7] Söderman A-CH, Odhagen E, Ericsson E et al (2015) Post-tonsillectomy haemorrhage rates are related to technique for dissection and for haemostasis. An analysis of 15734 patients in the National Tonsil Surgery Register in Sweden. Clin Otolaryngol 40:248–254. 10.1111/coa.12361 25515059

[8] Bugten V, Wennberg S, Amundsen MF, Blindheimsvik MAB (2022) Reducing post-tonsillectomy haemorrhage: a multicentre quality improvement programme incorporating video-based cold technique instruction. BMJ Open Qual 11:e001887. 10.1136/bmjoq-2022-001887 36410782 PMC9680151

[9] Cushing H (1928) Electrosurgery as an aid to the removal of intracranial tumors with a preliminary note on a new surgical current generator by W.T. Bovie. Surg Gynecol Obstet 47:751–784

[10] Nabili V, Koempel JA (2005) Electrocautery tonsillectomy: common practice since the 1930s? Otolaryngol Head Neck Surg 133:818–819 16274822 10.1016/j.otohns.2005.07.022

[11] Davidoss NH, Eikelboom R, Friedland PL, Santa Maria PL (2018) Wound healing after tonsillectomy - a review of the literature. J Laryngol Otol 132:764–770 30289104 10.1017/S002221511800155X

[12] Leinbach RF, Markwell SJ, Colliver JA, Lin SY (2003) Hot versus cold tonsillectomy: a systematic review of the literature. Otolaryngol Head Neck Surg. 10.1016/S0194-5998(02)00729-0 14574289

[13] Howitz MF, Vilhelmsen J, Wennervaldt K, et al (2023) Tonsillectomy and risk of post-Tonsillectomy and risk of post-tonsillectomy haemorrhage-a tonsillectomy haemorrhage-a protocol for a randomised clinical trial protocol for a randomised clinical trial 37341351

[14] Danish National Health Agency (2016) National klinisk retningslinje for fjernelse af mandler (tonsillektomi), 1st ed. Danish National Health Agency 2016

[15] Harris PA, Taylor R, Minor BL et al (2019) The REDCap consortium: building an international community of software platform partners. J Biomed Inform. 10.1016/j.jbi.2019.103208 31078660 PMC7254481

[16] Harris PA, Taylor R, Thielke R et al (2009) Research electronic data capture (REDCap)-a metadata-driven methodology and workflow process for providing translational research informatics support. J Biomed Inform 42:377–381. 10.1016/j.jbi.2008.08.010 18929686 PMC2700030

[17] Wulu JA, Chua M, Levi JR (2019) Does suturing tonsil pillars post-tonsillectomy reduce postoperative hemorrhage? A literature review. Int J Pediatr Otorhinolaryngol 117:204–209 30611028 10.1016/j.ijporl.2018.12.003

[18] Senska G, Schröder H, Pütter C, Dost P (2012) Significantly reducing post-tonsillectomy haemorrhage requiring surgery by suturing the faucial pillars: a retrospective analysis. PLoS One 7:e47874. 10.1371/journal.pone.0047874 23118902 PMC3485309

[19] Abdelaty Elkholy T (2016) Modified Surgical Technique with Pillars Repair in Reducing Post Tonsillectomy Haemorrhage

[20] Seyhun N, Dizdar SK, Çoktur A et al (2020) Risk factors for post-tonsillectomy hemorrhage in adult population: does smoking history have an impact? Am J Otolaryngol. 10.1016/j.amjoto.2019.102341 31732315

[21] Nguyen TBV, Chin RY, Paramaesvaran S, Eslick GD (2014) Routine tonsillar bed oversew after diathermy tonsillectomy: does it reduce secondary tonsillar haemorrhage? Eur Arch Otorhinolaryngol 271:3005–3010. 10.1007/s00405-014-3075-6 24792067

[22] Coordes A, Soudry J, Hofmann VM, Lenarz M (2016) Gender-specific risk factors in post-tonsillectomy hemorrhage. Eur Arch Otorhinolaryngol 273:4535–4541. 10.1007/s00405-016-4146-7 27328963

[23] Windfuhr JP, Verspohl BC, Chen Y-S et al (2015) Post-tonsillectomy hemorrhage–some facts will never change. Eur Arch Otorhinolaryngol 272:1211–1218. 10.1007/s00405-014-3025-3 24737054

[24] Lowe D, van der Meulen J, National Prospective Tonsillectomy Audit (2004) Tonsillectomy technique as a risk factor for postoperative haemorrhage. Lancet 364:697–702. 10.1016/S0140-6736(04)16896-7 15325834

[25] Akin RC, Holst R, Schousboe LP (2012) Risk factors for post-tonsillectomy haemorrhage. Acta Otolaryngol 132:773–777. 10.3109/00016489.2012.660545 22568500

[26] Mueller J, Boeger D, Buentzel J et al (2015) Population-based analysis of tonsil surgery and postoperative hemorrhage. Eur Arch Otorhinolaryngol 272:3769–3777. 10.1007/s00405-014-3431-6 25502742

[27] Riggin L, Ramakrishna J, Sommer DD, Koren G (2013) A 2013 updated systematic review & meta-analysis of 36 randomized controlled trials; no apparent effects of non steroidal anti-inflammatory agents on the risk of bleeding after tonsillectomy. Clin Otolaryngol 38:115–129. 10.1111/coa.12106 23448586

[28] Hoshino T, Tanigawa T, Yanohara G et al (2017) Effect of body mass index on posttonsillectomy hemorrhage. Biomed Res Int 2017:9610267. 10.1155/2017/9610267 28555197 PMC5438846

[29] Østvoll E, Sunnergren O, Ericsson E et al (2015) Mortality after tonsil surgery, a population study, covering eight years and 82,527 operations in Sweden. Eur Arch Otorhinolaryngol 272:737–743. 10.1007/s00405-014-3312-z 25274044

[30] Faramarzi M, Shishegar M, Kazemi T et al (2021) The effect of applying amniotic membrane on post-tonsillectomy pain and bleeding. Eur Arch Otorhinolaryngol 278:485–492. 10.1007/s00405-020-06173-7 32601919

[31] Cetiner H, Cavusoglu I, Duzer S et al (2017) Effect of suturation plus Surgicel application on post-tonsillectomy bleeding and pain. J Craniofac Surg 28(7):e672. 10.1097/SCS.0000000000003827 28857996

[32] Stalfors J, Ovesen T, Bertelsen JB et al (2022) Comparison of clinical practice of tonsil surgery from quality register data from Sweden and Norway and one clinic in Denmark. BMJ Open 12:e056551. 10.1136/bmjopen-2021-056551 35477880 PMC9047789

[33] Sunnergren OAFAS et al (2021) Kalla-tekniker för såväl dissektion som blodstillning bör användas vid tonsillektomi. [Cold techniques for both dissection and hemostasis should be used foor tonsillectomy.]. ÖNH-Tidskrift 2:12–14

[34] Engesæter I, Bugten V, Wennberg S et al (2025) Postoperative bleeding after tonsillectomy–a risk factor study on 28,254 patients. Acta Otolaryngol. 10.1080/00016489.2025.2561903 40996221

[35] Alenezi MM, Al-Harbi FA, Almoshigeh ANM et al (2025) Comparison of post-tonsillectomy hemorrhage rate after different tonsillectomy techniques: systematic review and meta analysis. Clin Pract. 10.3390/clinpract15050085 40422266 PMC12110027

